# We’re only human after all: a critique of human-centred AI

**DOI:** 10.1007/s00146-024-01976-2

**Published:** 2024-06-04

**Authors:** Mark Ryan

**Affiliations:** Wageningen Economic Research, Wageningen University and Research, Droevendaalsesteeg 4, 6708 PB Wageningen, The Netherlands

**Keywords:** Foucault, Philosophy of technology, Human-centred artificial intelligence, Ethics of AI, The Order of Things

## Abstract

The use of a ‘human-centred’ artificial intelligence approach (HCAI) has substantially increased over the past few years in academic texts (1600 +); institutions (27 Universities have HCAI labs, such as Stanford, Sydney, Berkeley, and Chicago); in tech companies (e.g., Microsoft, IBM, and Google); in politics (e.g., G7, G20, UN, EU, and EC); and major institutional bodies (e.g., World Bank, World Economic Forum, UNESCO, and OECD). Intuitively, it sounds very appealing: placing human concerns at the centre of AI development and use. However, this paper will use insights from the works of Michel Foucault (mostly *The Order of Things*) to argue that the HCAI approach is deeply problematic in its assumptions. In particular, this paper will criticise four main assumptions commonly found within HCAI: human–AI hybridisation is desirable and unproblematic; humans are not currently at the centre of the AI universe; we should use humans as a way to guide AI development; AI is the next step in a continuous path of human progress; and increasing human control over AI will reduce harmful bias. This paper will contribute to the field of philosophy of technology by using Foucault's analysis to examine assumptions found in HCAI [it provides a Foucauldian conceptual analysis of a current approach (human-centredness) that aims to influence the design and development of a transformative technology (AI)], it will contribute to AI ethics debates by offering a critique of human-centredness in AI (by choosing Foucault, it provides a bridge between older ideas with contemporary issues), and it will also contribute to Foucault studies (by using his work to engage in contemporary debates, such as AI).

## Introduction

Research on the ‘human-centred’ artificial intelligence approach (HCAI) has substantially increased over the past few years. A quick search on Scopus will return 1657 results mentioning HCAI, with most of them published since 2019.^1^ At least 27 universities have established labs, institutes, or research centres promoting an HCAI approach, such as the Stanford Human-Centered AI Institute, the Berkeley Center for Human-Compatible AI, the Chicago Human AI Lab, the Utrecht Human-centered AI, and the University of Technology Sydney’s Human-Centric AI Centre (Human-Centered AI 2023; Shneiderman 2022). HCAI is also being adopted by NGOs, civil society organisations, professional organisations, and affiliations (Human-Centered AI 2023; Shneiderman 2022) and large tech companies [such as IBM Research (2021); Google (2023); and Microsoft (2023)].

Human-centred or human-centric approaches to AI have also been strongly adopted in recent political discourse, particularly in Europe. One of the most important sets of AI ethics guidelines, the High-level Expert Group’s (HLEG) Ethics Guidelines for Trustworthy AI, states that AI should ‘be human-centric’ (p. 8). This human-centric approach provides the basis for the EU AI Act, which President of the European Commission Ursula von der Leyen has recently confirmed: ‘Our AI Act will make a substantial contribution to the development of global rules and principles for human-centric AI’ (European Commission 2023a). Overall, the EU views the development and promotion of human-centredness as a cornerstone to the success of AI in Europe (European Commission 2021, 2024a, 2024b).

HCAI is also a part of many transnational organisations' core principles, such as the OECD (OECD 2019). The OECD Principles for Trustworthy AI, of which HCAI is one of the core components, have been adopted in over 50 countries (Russo and Oder 2023). HCAI has also been used as the guiding principle in documents by many powerful groups and institutions, such as the World Economic Forum (World Economic Forum 2021), the G7 (European Commission 2023b), the G20 (2019), the United Nations (2024), the World Bank (2020), and UNESCO (2018).

Those advocating for HCAI claim that AI is currently being designed with the goal of technological progress while overlooking its impact on humans (Ozmen Garibay et al. 2023, p. 392). HCAI proponents state we should move away from being technology-focused to human-focused (Bingley et al. 2023). We must ‘re-position humans at the center of the AI lifecycle’ (Ozmen Garibay et al. 2023, p. 393). AI should be based on human needs (Hartikainen et al. 2022) and amplify, enable, and benefit human capabilities (Pacailler et al. 2022). AI should be designed to ‘enhance humanity and suit their interests’ (Mhlanga 2022, p. 12).

Those writing on HCAI focus on bringing together human beings and AI together to work alongside one another to ensure human progress (Hartikainen et al. 2022). HCAI will improve interactions and relationships between humans, intelligent robots, and AI systems (He et al. 2021). It is needed to ‘counter the earlier fears and anxieties that artificial intelligence automates everything, replaces and displaces humans, and pushes them into passive roles’ (Holzinger et al. 2022, p. 4). We need to place humans as the centre of AI development, deployment, and use to create technology that ‘supports humanity’ (Cherrington et al. 2020, p. 4).

This paper evaluates 20 HCAI texts to identify some common themes and positions within this approach. This paper applies the work of Michel Foucault (in particular, The Order of Things) to critically engage with some of the main suppositions found within HCAI literature.

While several researchers have written about Foucault and *technology* (Barn and Barn 2018; Bergen and Verbeek 2021; de Laat 2019; Dorrestijn 2012a, b, 2017; Hernández-Ramírez 2017; Laes and Bombaerts 2021, 2022), very little has been written specifically on the topic of *AI*. In addition, most of these texts focus on Foucault’s later work [e.g., on Technologies of the Self and the History of Sexuality (Foucault 1988b, 1990)], and less on his earlier work, such as *The Order of Things*.^2^

This paper will contribute to the field of philosophy of technology by using Foucault's analysis to examine assumptions found in HCAI (it provides a Foucauldian conceptual analysis of a current approach (human-centredness) that aims to influence the design and development of a transformative technology (AI)). It will contribute to AI ethics debates by offering a critique of human-centredness in AI (by choosing Foucault, it provides a bridge between older ideas and contemporary issues), and it will also contribute to Foucault studies (by using his work to engage in contemporary debates, such as AI). Overall, this paper demonstrates that certain areas of knowledge (e.g., AI) make problematic assumptions that were challenged decades ago (e.g., by Michel Foucault), such as the way scientists and technologists conceptualise the human.

This paper is divided into three sections: Sect. 2 provides a very brief outline of Michel Foucault, giving a broad background of his thoughts and how they relate to the human. Section 3 outlines how his insights are relevant for analysing five main assumptions found within HCAI: human–AI hybridisation is desirable and unproblematic (3.1); that humans are not currently the centre of the AI universe (3.2); that we should use the human as a way to guide AI development (3.3); AI is the next step in a continuous path of human progress (3.4); and increasing human control over AI will reduce harmful bias (3.5). Section 4 is the conclusion of the paper.

## Michel Foucault and the human

In *The Order of Things*, Foucault examines who we are by looking at the developments in thought and practice and how particular power relationships have affected them (Foucault 1994). He claims that our history has undergone major transformations in knowledge and thought, through three shifts or junctures, or what he calls ‘epistemes’ (the Renaissance,^3^ the classical period,^4^ and modernity) (Foucault 1994). Epistemes are the underpinning conditions of possibility that give rise to a particular epistemological moment in history. They cause, create, and shape the very ‘structures of knowledge to emerge in a given cultural period and at a given moment’ (Downing 2008, p. 39).

Knowledge does not appear or result linearly but is caused by many epistemic changes and ruptures throughout history (Downing 2008, p. 39). The epistemic field is what allows the creation of certain ideas and thoughts at a given time (Downing 2008, p. 41). The epistemes are the ways that we envision the world, not from a particular discipline or belief system, but what underpins and allows particular ways of viewing the world to materialise in the first place (Foucault 1994):

Foucault wishes to establish here a history that looks deeper than individual experience or consciousness, and that questions our assumption that we are uniquely aware of, or in control of, the decisions we make: rather than having at our disposal an infinite world of thinkable possibilities, we are limited by our own–invisible–epistemic moment and its contingent rules (Downing 2008, p. 40).

Foucault analyses modes of thinking within certain events and practices, and the ‘investigation no longer concerns what the individual undergoes; it concerns the historical conditions of their undergoing it’ (May 2014, p. 23). Foucault does not examine particular events to get a better account or framing of these practices, but instead, to gain insights on the historical frameworks in which they are embedded and have given shape to, and tell us about, who we are today (May 2014, p. 23):

what I am attempting to bring to light is the epistemological field, the episteme in which knowledge, envisaged apart from all criteria having reference to its rational value or to its objective forms, grounds its positivity and thereby manifests a history which is not that of its growing perfection, but rather that of its conditions of possibility; in this account, what should appear are those configurations within the space of knowledge which have given rise to the diverse forms of empirical science (Foucault 1994, p. xxii).

The focus of this paper will be on the modern episteme, which began around the end of the 18th and beginning of the nineteenth century. The modern episteme was the beginning of when ‘man’ (human)^5^ became both the object and subject of analysis (Foucault 1984a, b, c, p. 35).^6^ The human is both the observed (object) and observer (subject), which appeared as a form of self-knowledge created and encapsulated through the emergence of the human sciences. However, Foucault is not referring to the human as the species homo sapiens but is instead referring to a privileged moment in history where we are both the subject and object of investigation. Essentially, he refers to the human as a being that can analyse itself while creating and defining what oneself is (Foucault 1994).^7^

One of the most striking features of the modern episteme is that it supports a critical evaluation and relationship with one’s present (May 2014, p. 22). Thought in the modern episteme attempts to grasp humankind’s place in the world, be responsive to it, evaluate it, test it, and experiment with it in an almost ‘permanent critique of our historical era’ (Foucault 1984c, p. 312).^8^ We are deeply engrained in our history, and it defines who we are (May 2014, p. 11). Our history has led to this particular place where we are situated, which could have been different, and would have also led to a different outcome in who we are now: ‘we are this rather than that as a matter of contingency, not necessity. We did not have to be this rather than that, which means, among other things, that we do not have to continue to be this’ (May 2014, p. 11). We are the result of a contingent history and this history has shaped who we are today (Foucault 1994). However, our history could have taken different paths, we could have responded differently at different times, or it could have had different junctures than what it did (May 2014, p. 15).

In the context of AI, our knowledge, ability, and skills go into the development of AI, based on the human way of experiencing the world. While AI is being used in a wide diversity of applications, much revolves around analysing human or human activities (while AI can be applied in other circumstances, the application to humans and human activities is the primary focus of this paper) (Watters 2023). While Foucault wrote before the advent of high-speed internet, advanced digitalisation, and the complex developments of robotic technology, his work offers fruitful insights into the AI debate. The re-examination of Foucault’s work in such a way provides new insights into contemporary issues (such as AI), while also building upon the ideas developed in Foucault’s work.^9^ This paper will use a conceptual analysis of HCAI using Foucault’s writings, focusing on his critique of the human in The Order of Things. The paper uses literature on HCAI that provides information about this approach (see Table 1).

**Table 1:** HCAI literature review process

HCAI literature review process	
The following query was inputted into Scopus on February 1st 2023: (TITLE-ABS-KEY ("human-centred AI") OR TITLE-ABS-KEY ("human-centered AI") OR TITLE-ABS-KEY ("human-centric AI") OR TITLE-ABS-KEY ("human-centred artificial intelligence") OR TITLE-ABS-KEY ("human-centered artificial intelligence") OR TITLE-ABS-KEY ("human-centric artificial intelligence"))	
It resulted in 334 results, of which 314 were excluded because they were not explicitly about human-centred AI (149), did not provide much information about the values or content of the HCAI approach (114), or because they had no author, were duplications or were unretrievable (51)	
This left 20 articles for analysis	

This paper used these 20 articles to provide the basis for a critical reflection on the positions found within HCAI literature. In particular, this paper will focus on four prominent claims that were commonly found within the HCAI literature: human–AI hybridisation is unproblematic and desirable (Sect. 3.1.); the human is not already at the centre of the AI universe (Sect. 3.2.); humans *should* be at the centre of the AI universe (Sect. 3.3.); AI is the next step in a continuous path of human progress (Sect. 3.4.); and increasing human control over AI will reduce harmful bias (Sect. 3.5.).

## Examining HCAI through the lens of The Order of Things

It is important to note that the views found within HCAI are not in themselves new and appear to echo earlier views found in many different user-focused and design approaches; many of which have also been incorporating AI into their most recent formulations. For example, Human–Computer Interaction (HCI) (and its various “waves”) (Choudhury et al. 2020; Li and Hilliges 2021; Nazar et al. 2021), Interaction Design (Liu 2022; Zhang and Jia 2021), and Human-Centred Design (Cherrington et al. 2020; Margetis et al. 2021; Rantavuo 2019). Two early pioneers in this community were Licklider (1969) and Engelbert (2003), both of whom influenced the work of the most influential HCAI scholar to data, Ben Shneiderman (2022).^10^ The co-development of human–machine interactions was advanced by Licklider (1960) and Engelbart (1962/2003), both of whom were also influenced by earlier research on human-technology symmetry (see Bush, 1945, and Kapp’s view of technologies as projections of human organs (1877/2018)).

There have been many different threads of thought and waves within HCI (Bødker 2006; Grudin 2005), but generally, they focus on how ‘man–machine interfaces’ should be designed for ‘reducing training time, but most important was eliminating errors and increasing the pace of skilled performance’ (Grudin 2005, p. 47). Despite this, Grudin (2009) argued that HCI was not prepared to respond to AI because of the fast pace at which AI is developing and because of the uncertainty of the role of the human agent in the process. Therefore, HCI had to develop or respond to the challenges presented by AI, which is perhaps one reason for the emergence of HCAI.

Besides HCI, it may be proposed that HCAI also stems from the approach of ‘human-centred design’. Human-centred design is an ‘approach to interactive systems development that aims to make systems usable and useful by focusing on the users, their needs and requirements, and by applying human factors/ergonomics, and usability knowledge and techniques’ (ISO 9241–210:2019(E)). It aims to incorporate human values earlier in the design process of technologies and to emphasise the importance of the social sciences (Hanington 2010). The HLEG defines it similarly, with the integration of human values being central to AI development and use: ‘The human-centric approach to AI strives to ensure that human values are central to the way in which AI systems are developed, deployed, used and monitored (High-Level Expert Group on AI 2019, p. 37). While human-centred design has been extensively discussed by many scholars (see Hanington 2010; Norman 1988/2013, Norman and Draper 1986), human-centred *AI* has been much less discussed, with little to no critical evaluation of it being done.

There is an influential lineage of thought that refers to human–machine dynamics, where HCAI is the next step in this process. While this paper is not necessarily a critical examination of these other positions (human-centred design and HCI, for example), the criticisms directed against HCAI in the following sections may also be relevant to many of these other approaches. The following sections will concentrate on HCAI and issues within this approach when analysed through the lens of Foucault’s work, with a specific focus on his critique of the human in *The Order of Things*.

### What is human? Human–AI hybridisation is desirable and unproblematic (Claim 1)

HCAI researchers advocate a human–machine hybrid intelligence or a human–machine system that is better than either one alone, with the end goal of a human–machine symbiotic world (Xu et al. 2022) (such a view stems from a long lineage of thinking about the hybridisation of human–machine intelligence, most notably (Engelbart 1962; Licklider 1969). It is a ‘symbiosis of human and artificial intelligence’ (Horvatić and Lipic 2021, p. 2). This symbiosis is a way to ‘improve’ human abilities: ‘With artificial intelligence, we have the chance to create a change for improving the abilities of the mind, similar to what engines and motors have done for the body’ (Schmidt 2020, p. 4).

HCAI researchers claim that we should ‘augment humans and human intelligence’ (He et al. 2021, p. 11). Human intelligence can be extended through the use of AI (Mhlanga 2022). Essentially, we should combine AI and ‘natural’ intelligence (which HCAI anthropocentrically limits only to *human intelligence*) ‘to empower, amplify, and augment human performance, rather than replace people’ (Holzinger et al. 2022, p. 1). AI should not replace ‘natural [human] intelligence’, but instead, it should be used to augment it, ‘like “power steering for the brain”’ (Holzinger et al. 2022, p. 2). There is pressure on individuals to hybridise themselves because ‘[n]ot using AI will create major disadvantages, as others will be faster, see things you do not see, and will be able to predict events long before you encounter them’ (Schmidt 2020, p. 4). Essentially, AI should ‘amplify, augment, empower, and enhance human performance’ (Shneiderman 2022, p. 4).

The hybridisation discussed in HCAI concerns intellectual and physical human capacities (Schmidt 2020, p. 4) However, this raises several questions about what is, and will be, constituted as ‘human’ and whether this will change due to this hybridisation. Will these hybrid human AIs be more or less human than humans who do not adopt and integrate AI in such a way? If we use ‘human’ to centre AI development, how will those who do not hybridise themselves be viewed and valued?

I believe Foucault’s work can offer some insights on this matter. Foucault stated that within the broad history of our species, the ‘human’ is a relatively ‘recent invention’. In the past, subjects have been referred to as mortals, serfs, vassals, denizens, slaves, and subjects of the emperor. The human as a subject has only really emerged as an important subject since the modern episteme (Foucault 1994, p. 308).^11^ The modern human has been so inundated and ‘blinded by the recent manifestation of man that we can no longer remember a time – and it is not so long ago—when the world, its order, and human beings existed, but man did not’ (Foucault 1994, p. 322). Despite this, there is a chance that the human is a ‘historically contingent construct’, which, in the future, will be ‘erased, like a face drawn in sand at the edge of the sea’ (Foucault 1994, p. 387). When Foucault calls man a recent invention, he is describing the emergence of the subject as a human:

To be sure we often do use the term ‘human’ to describe subjects in ancient or medieval times, but they would not think of themselves as ‘human’ in the way that we employ this term with all of its biological, linguistic and other ontological trappings. They might conceive themselves as mortals, or as entrapped souls waiting for judgment etc., but this is a far cry from suggesting that these historical subjectivities are merely different modes of one common, though vague conception of humanity (Lightbody 2010, p. 25).

Therefore, the subject has had many different guises over the centuries, and the human is simply the latest. The human is a recent invention that may pass away like other historical entities, when ‘something new will arise’ (Foucault 1994, p. 54). Foucault is not describing the *literal* death of humankind, but is speaking figuratively: ‘It is obvious that in saying […] that man has ceased to exist I absolutely did not mean that man, as a living or social species, has disappeared from the planet’ (Foucault and Carrette 2013, p. 101). He was also only describing the *possible* (and not inevitable) death of the modern human (Sutherland and Patsoura 2015, p. 295).

Foucault’s understanding of the human may offer insights into the belief in HCAI of a human–AI hybridisation. This hybridisation of humans and AI will affect what we define and classify as human, which may also impact what we *value* as being human. If we evaluate human well-being as the goal of HCAI, human–AI hybridisation confuses what is or remains ‘human’ when this hybridisation increases. As well as being practically problematic, this human–AI hybridisation raises serious ethical questions about degrees of humanness. It may reduce the value of those who cannot or do not want to hybridise with AI if we use the human–AI hybrid as our paradigm of the human and the goal of AI development (criticisms of human-technology hybridisation have frequently been discussed in cyborg and transhumanist literature, cf. Clark 2001; Haraway 2000; Hayles 2000). The non-human, or lesser-than-human, may either be the new AI-human hybrids or those who refuse to or cannot hybridise.

An issue with AI-human hybridisation and redefinition of the human is that the human is not simply a descriptor but also an inherently normative inflection (Lightbody 2010). The answer to ‘What is human?’ has allowed many interpretations to emerge over the previous centuries, which has at times resulted in the exclusion and disenfranchisement of certain groups (Lightbody 2010). For Foucault, when we define human, we must also describe what is non-human or other, that which ‘is inherently different, dangerous, and, perhaps, evil’ (Lightbody 2010, p. 21). Therefore, defining the human may lead to suffering and abuse of certain people and groups defined as inhuman or other than human (Lightbody 2010). Using a definition of human and non-human has been a tool to create tension between people, and oppressed groups and even as a way to excuse genocide: ‘During the Holocaust, Nazis referred to Jews as rats. Hutus involved in the Rwanda genocide called Tutsis cockroaches. Slave owners throughout history considered slaves subhuman animals’ (Talk of the Nation 2011).

While these are extreme examples of how distinguishing between the human and non-human can be problematic, their relevance for the distinction required in HCAI is still essential. If we are to adopt the HCAI belief that the hybridity between humans and AI will blur the lines of what is and is not human, this opens the possibility of creating these kinds of tensions and divisions among people and groups. In the extreme examples given above, the other is seen as less than human, where the human has a reduced, or devoid, moral obligation towards them. Definitions of the human and the non-human have been used throughout history to justify violence, oppression, and hatred by one group over another (Smith 2011, 2020, 2021). Essentially, if we endorse HCAI’s AI-human hybridity proposal becomes true, there is a distinct possibility that this hybridity will blur the boundaries of what is (or is not) considered human.

Not only is this problematic from a practical standpoint (the human becomes blurred in the AI-human hybrid), but it is also questionable from an ethical standpoint (it may lead to the oppression and abuse of those considered less than human, as a result of the new distinctions created by AI-human hybridisation).

### The human is not at the centre of the AI universe (Claim 2)

The previous section demonstrated that using humans as the goal of AI development is problematic if we acknowledge the HCAI goal of human–AI hybridisation. What is or is not human becomes challenging to identify due to increasing levels of AI hybridisation. As a result, this may also have normative implications about who is valued or valued more due to this changing definition of the human.

Apart from these concerns, HCAI proponents make another problematic claim: humans are currently *not* at the centre of AI development. For example, one of the leading proponents of HCAI, Ben Shneiderman, claims that we must implement a ‘Second Copernican Revolution’ to place humans at the centre of AI development (Shneiderman 2020). Shneiderman states that most people viewed Earth as the centre of the solar system before the Polish scientist Nicolaus Copernicus demonstrated that it is the sun.^12^ Shneiderman uses the Copernican Revolution and applies this as an analogy to AI. He claims that algorithms are currently the centre of AI development. However, we should place humans at the centre of the AI universe, in a similar way that Copernicus put the sun (and not Earth) as the centre of the universe (Shneiderman 2020).

Using the analogy of the Copernican Revolution as a way to place humans at the centre of the universe appears somewhat counterintuitive because it is usually understood as a metaphor to demonstrate that ‘[n]o longer was humanity at the centre of the universe, about which all else revolved, but rather humanity was but one small part of a much larger system in constant movement’ (Hornsby 2023). The metaphor of the Copernican Revolution has often been used to point out the flaws of anthropocentrism and the misguided placement of ourselves at the centre of the universe (Freud 2021; Kuhn 1957; Sagan & Druyan 2011).^13^ However, Shneiderman uses the Copernican Revolution, which is often used as a metaphor for greater human humility and understanding of our limited place on the planet, to place humans at the centre of the (AI) universe (Shneiderman 2020).

Regardless of the striking contradictions with using the Copernican Revolution as a metaphor for AI development, Shneiderman’s position—that humans are not at the centre of AI development—is a core theme throughout HCAI literature. This type of human-centric attitude is what Foucault was often critical of. Foucault pointed out that examples such as the Copernicus revolution should work as moments to realise our limited place in the universe and allow us to emancipate ourselves from the binds of our self-importance: ‘We are inclined to believe that man has emancipated himself from himself since his discovery that he is not at the centre of creation, nor in the middle of space, nor even, perhaps, the summit and culmination of life’ (Foucault 1994, p. 348).

Unfortunately, it appears that we have not emancipated ourselves from ourselves but rather built artificial constructs that think, act, and speak like us while also claiming that these entities are (in fact) at the centre of the universe and not us. Furthermore, instead of self-reflecting on this possibility, the HCAI approach states that we need to (re)claim our place at the centre of the AI universe before it is too late. Those defending this view state that the field of AI is too stringently focused on developments, advances, and technological ingenuity, and those working in the field often become distracted from the needs of humans (this criticism against the lack of including the human perspective from computer development stems from HCI. See Bødker and Iversen (2002); Bødker (2006); Göransdotter and Redström; Höök and Löwgren 2021).

HCAI proponents state that AI development ‘needs to focus much more on humans than it currently does’ (Bingley et al. 2023, p. 6). This perspective assumes that the human is not (currently) the centre of AI development and that technology or algorithms are at the centre of a ‘technical, machine-centred approach, with little focus on the human’ (Mhlanga 2022, p. 10).^14^ When analysing this view through the lens of Foucault’s work, this HCAI position would appear misguided. This is because Foucault stated that the human has fundamentally been the centre of our scientific analysis in the modern episteme—throughout which the many human sciences are illustrative of this (Foucault 1994). The study of the human in the modern episteme is more important than any other object of concern in earlier epistemes, such as Truth, God, and Logos (Downing 2008, p. 45). The human of the modern episteme is ‘a being such that knowledge will be attained in him of what renders all knowledge possible’ (Foucault 1994, p. 318). What differentiates the modern episteme’s analysis of the human is that it proposes that the study of the human differs from the analysis of other objects of scientific analysis. Fundamentally, in the modern episteme, the human receives special attention in the hierarchy of observable things (Maniglier 2013).

Despite this, HCAI researchers claim that AI development does not place the human at the centre.^15^ If this view of HCAI is correct, then AI is being developed with some other goal in mind or the interests of something other than humans. If the human is not at the centre, then who or what *is* at the centre (for example, human-centred designers earlier claimed what is at the centre is the process or function of the technology’s design, rather than the human, cf. Hanington 2010)?^16^

Instead of proposing that some other species or entity is the primary focus of AI development, perhaps HCAI researchers are claiming that the values that guide machine-centred approaches are more likely to cause harm than those found within HCAI. Or that the values of HCAI are somehow better than those of the machine-centred approach (this is a similar claim made in HCI, interactive design, and user-centred design, from which HCAI appears to take influence; cf. Göransdotter, M and Redström, Höök and Löwgren 2021). Therefore, we should prioritise *human-centred values* over *machine-centred values*.

If HCAI researchers endorse this, then they would have to demonstrate that the values implicit within machine-centred approaches do not care about humans either *at all* or *enough*. Humans are not at the centre of current AI development, and thus, we need human-centred AI to replace it (also reflected in earlier human-centred design approaches, cf. Göransdotter and Redström; Hanington 2010). Proposing that current values in AI are not human-centred *at all* is a bold claim and would require showing that ‘machine-centred’ values completely disregard humans. One way to do this would be to outline machine-centred values and demonstrate how they are against humanity. As there does not appear to be anyone that calls themselves ‘machine centred’ (a term made up by HCAI advocates),^17^ we need to intuit the values this position *may* support (e.g., technological ingenuity and scientific innovation). While one may claim that these values sometimes lead to harm towards humans, this does not necessarily make them against humanity (similarly, values endorsed in HCAI may also cause harm and benefit, depending on how they are interpreted and implemented). Assuming that HCAI advocates do not want to go down this route, this paper will evaluate the less incredulous proposition that current values in AI development are not human-centred *enough*.

To do this, HCAI proponents would have to demonstrate how certain values are more human-centred and other values are less human-centred. While values have changed throughout history and different values have come to replace others, basing our prioritisation of values on the foundation that one set of values is more ‘human’ than others appears challenging. This is because values change and are a collective activity within society, rather than something fixed. If one attempts to classify certain values as being more human than others, one would need a distinct understanding of what it means to be human. To distinguish what values are more or less human, one needs a core meaning of the human on which to identify how values are closer to or further away from this core humanness. To do this, one would need some core definition of the human, something like the idea of ‘human nature’, which Foucault was also critical of in other works (see Foucault 1988a, p. 12).

A distinct challenge with distinguishing core human values based on an idea such as human nature is that what is considered good now and the values we accept in society regularly change. To distinguish certain values as being more human than others, as if they are fixed, perhaps with some idea of what ‘human nature’ is, is very difficult (Foucault 1988a, p. 12). In response to this, Foucault claims that because our understanding of human nature is constantly changing and diverse, trying to base human values on it is deeply problematic (Foucault 1994).

In addition to these issues, basing human values on a definition of human nature may lead to a form of ‘ethnocentrism’ (this is where specific populations, cultures, or ethnicities, claim that their values are more human or humane than others) (Smith 2011). If one claims that one’s values are more human than another’s, or one’s values have achieved a greater level of moral development than another, this may imply that the values of the ‘other’ are ‘barbarian’, ‘primitive’, or generally, less than one’s own (Smith 2011). This type of attitude often results in a lack of acceptance and respect for diversity and the oppression and marginalisation of other groups (Smith 2011). Therefore, ethnocentrism may also lead to a type of cultural imperialism, where one group (the one whose values are ‘superior’) imposes their values on another because their values are more human (many have written on cultural imperialism, cf. Appiah 2017; Freire 2018; Said 1977, 2012; Tomlinson 2001).

Therefore, the claim that AI is not being developed with the human at the centre is misleading and ethically problematic. There is no clear evidence to demonstrate that machine-centred approaches do not care about humans at all or enough. While HCAI proponents disagree with the values that machine-centred approaches prioritise, this does not necessarily mean they are *less human* than those HCAI endorses.

### We *should* place humans at the centre of AI development (Claim 3)

As was shown in Sect. 2.1., HCAI’s definition of the human is unclear, particularly, when applying their AI-human hybridisation model. Furthermore, HCAI researchers’ claim that humans are not already the centre of our concern is also problematic, both from a practical perspective and an ethical perspective. HCAI proponents also make the normative claim that we should place humans at the centre of AI development. The human is both subject and object of the knowledge and decisions generated by AI, and this is justification for why humans *should* be placed at the centre: ‘humans should be placed at the centre of the discussion as humans are ultimately both the actors and the subjects of the decisions made via algorithmic means’ (Lepri et al. 2017, p. 6).

However, Sect. 2.1 demonstrated that if we adopt HCAI’s belief that the humans will be hybridised with AI, then the definition of human becomes blurred. Not only could this lead to practical difficulty in identifying what is human, but it is also ethically problematic. For example, people who choose not to hybridise (or those who do) may be viewed as less than human. Therefore, placing humans at the centre of our concerns in AI is difficult to normatively defend based on the account that the human is the main subject and object of algorithmic decision-making alone.

However, there may be *other* reasons for justifying HCAI’s placement of the human as the centre. For example, there is a claim that we should place human at the centre to escape the Western-dominated, colonising approach found within AI. Some HCAI advocates claim that AI is Western-dominated and overlooks other cultures (Mhlanga 2022; Murphy and Largacha-Martínez 2022):

AI is portrayed, as an idealized rendition of rationality, other modes of knowledge are overwhelmed. Consistent with the Western penchant for searching for ultimate foundations—Ideas, God, natural laws, etc.—algorithms have been given a seignorial status (Murphy and Largacha-Martínez 2022, p. 1).

This Western attitude claims AI is a form of objective knowledge, while humans are emotional and flawed. This Western worldview assumes that AI can arrive at more objective decisions than those made by humans (Lepri et al. 2017). AI can perform tasks quicker than humans, process larger quantities of data, and it does ‘not get tired, hungry, or bored and they are not susceptible to corruption or conflicts of interests’ (Lepri et al. 2017, p. 1). The Western-dominated approach to AI claims that algorithms do not contain the flaws of human reasoning and thus, human knowledge should be made subservient to the rational advice of AI decision-making (Murphy & Largacha-Martínez 2022).

In response to this colonising attitude, some HCAI advocates claim that we need to place the humans at the centre of AI decision-making to ‘decolonise AI’ (Murphy and Largacha-Martínez 2022). However, the colonising attitude that these authors are trying to get away from is (actually) the exact colonising attitude that also underpins HCAI—namely, a rationalist understanding of humankind, which is grounded in Western ideology about the human’s place in the world. Murphy and Largacha-Martínez 2022 are attempting to get away from a biased, Western approach to AI, by implementing an approach that is inherently underpinned by this Western worldview (HCAI).

HCAI, at its core, is a Western rationalist ideology that views AI as something like stone, iron, and bronze, a raw material that will allow humans to develop and be used for human purposes (Schmidt 2020). HCAI proponents claim that the current challenges that we face in the world are simply obstacles that a rational human can overcome with the help of AI, as we have done throughout history (Ozmen Garibay et al. 2023, p. 391). However, it is only a certain type of human: one that welcomes a future of ubiquitous AI, or at least, one who does not resist it. Other cultures, nationalities, and religions have no choice but to accept, adopt, and integrate AI within every aspect of their lives and opting out is not an option. HCAI proponents are essentially deterministic about AI (as are most who are writing about AI for that matter), claiming that AI is here to stay and going back to a pre-AI world is not an option: ‘Opting out is like putting your head in the sand’ (Schmidt 2020, p. 4).

### AI is the next step in the continuous path of human progress (Claim 4)

In HCAI, progress is continuous, and AI, when used correctly, will help us advance and achieve our full human potential. AI is simply another tool that we should capitalise on for human progress. This type of attitude, particularly the view of ‘progress’, is something that Foucault was critical of in his work. While Foucault does not say that progress is not possible, he states that the idea of it being a processual and progressive thing, whereby, each age or epoch develops towards something, towards an advancement of humankind, is a simplification of our past and a negation of the tragic periods we have witnessed throughout history (Foucault 1972). This interpretation of progress claims that humanity is constantly progressing (for example, with and through AI), albeit with a few hiccups along the way (May 2014, p. 15).

Modernity demonstrates the *human* as the hero of our current situation and will discover all the secrets and truths in the world, allowing us to invent ourselves and produce who we want to be (Rabinow 1984, p. 42). In HCAI, the human is the hero that will bring us toward the future we desire, allowing us to ‘co-evolve’ alongside AI (Herrmann and Pfeiffer 2022, p. 12). The human is constantly developing and progressing to a more evolved state and any disturbance is seen as simply a glitch or deviation from where we are destined (Rabinow 1984, p. 39). This constant progress towards a more evolved state is something that *should* be developed and further exploited, albeit with a (re)emphasis on the importance of placing humans at the centre of AI development.

In contrast, Foucault attempted to show how history is *discontinuous* (Rabinow 1984, p. 49). History has ruptures, changes, and regressions, and it is not a ‘continuous path of progress toward the Truth’ (May 2014, p. 29). In the context of HCAI, Foucault’s work reflects that we should get away from the idea that if we simply implement greater human control (to the right degree) and automation (to the right extent), we will continue on the path to progress (Shneiderman 2022). We must be aware of the temptation that AI can bring us closer to the truth of everything, and that ‘one day we will know ourselves, that nothing will escape our [AI hybridised] intellectual gaze’ (May 2014, p. 29). We should be sceptical about the goal of placing humans at the centre of everything and that this will lead to desirable outcomes.

For Foucault, each historical period is not necessarily a development and refinement of the one that went before it (May 2014, p. 29). History is made of junctures and ruptures (Foucault 1972), and AI development and the placement of human at the centre of AI research (and AI research, generally) may not necessarily lead us closer to this ideal of progress and improvement that is promised in HCAI. Human-centring in AI development, and AI development itself, is not necessarily something we should aspire toward in the hope that it will bring us to a more enlightened state:

History is not of its nature progressive; it does not necessarily move from the more primitive to the more enlightened, from the barbaric to the civilized. History has neither telos—a goal—nor a structured movement. We can see this clearly if we abandon our commitment to historical continuity and allow ourselves to see breaks instead, moments in which one way of looking is replaced by another, not as an improvement on or a refinement of the earlier way, but simply as a new framework or perspective (May 2014, p. 35).

Foucault does not embrace the viewpoint that history is constantly progressive, nor does he view history and society in decline (Rabinow 1984). Likewise, if we view AI through a Foucauldian lens, AI will not help humanity achieve all of its wildest dreams nor is it focused on some kind of doomsday vision of AI superintelligence enslaving us. Instead, history flows and ebbs, sometimes taking sharp junctures that are either progressive or the opposite: ‘This does not *reverse* the assumption of historical progress; it *complicates* it’ (May 2014, p. 68). Historical progress is discontinuous with sudden transformations or accelerations, rather than the view that knowledge and human development are consistent and constant:

My problem was not at all to say, "Voila, long live discontinuity, we are in the discontinuous and a good thing too," but to pose the question, "How is it that at certain moments and in certain orders of knowledge, there are these sudden take-offs, these hastening of evolution, these transformations which fail to correspond to the calm, continuist image that is normally accredited?" (Foucault 1984a, p. 54).

AI should not simply be seen as another tool to fulfil the goal of human progress. AI also holds the possibility of causing serious ruptures in how we interpret the world, our basis for knowledge, and how we view ourselves. This fact has also been demonstrated in a recent PEW Research study of ‘979 technology pioneers, innovators, developers, business and policy leaders, researchers and activists’; where 37% said that AI will *not* make us better off by 2030 (Anderson and Rainie 2018). Therefore, even the experts developing, implementing, and policing AI are uncertain if it will lead to progress (Li and Du 2007).

These issues have also become evident in the recent open letter calling for the pause of large-scale AI experiments (more advanced than GPT4) for 6 months (Future of Life Institute 2023). This has received over 25,000 signatures^18^ and support from ‘Twitter CEO Elon Musk, Apple co-founder Steve Wozniak, and Pinterest co-founder Evan Sharp’ (Sivakumar 2023), as well as many pertinent figures that are developing (Bengio 2023) and investing in AI (Hogarth 2023).^19^ The general public also shares these concerns, with a recent survey showing that 46% of US adults were highly, or somewhat, concerned that AI will wipe out the human species (Bialik and Orth 2023).

Therefore, we *should* be sceptical that AI will lead us on a path toward progress. With such concern around AI, it is important to re-evaluate what we actually mean by progress and how AI can be incorporated into this vision if we want to incorporate it at all. Unfortunately, the answer is not so simple as signing a digital letter to freeze AI for 6 months or, the opposite, continue business as usual in the hope that AI companies will self-regulate themselves.

Foucault’s work may offer some valuable insights about this without necessarily providing a clear-cut solution. It is worth looking back to other junctures in history to look for insights into how analogous situations developed in the past and how these have impacted our present. Perhaps, we should look at certain practices, technologies, and applications that were banned or seriously limited in certain instances rather than only focusing on those that were permitted. Despite the deterministic view of many HCAI researchers and AI advocates generally, a future with ubiquitous AI is not necessarily inevitable and may not be completely desirable.

Foucault’s approach emphasises the importance of looking at history for insights into how we have come to be in our present, but also to ensure that we do not make the same mistakes of the past. We should try to understand our current practices based on an understanding of our history (Paden 1987). This is not meant as a way to return to, or strive towards, a relic from the past. It is meant as a way that informs us that there are different ways of living now, as there have been different ways of living in the past. The reason for examining history is to postulate who we are and who we might become (May 2014, p. 99).

The rules that we use to structure meaning and create order are not ‘inevitable, natural or—in any simple sense, ‘true’, but are wholly socially and historically constructed according to unconscious sets of governing rules’ (Downing 2008, p. 46). They stem from an incalculable number of consequences and interactions throughout history. Therefore, our knowledge, ideas, and beliefs are fundamentally dependent upon our particular place and time in history (Downing 2008, p. 39). We need to be aware of this and the impact our AI-related choices will have on the future. Instead of adopting the view that AI will solve the world’s challenges, we need to be more critical about how we define ‘progress’ and what this will mean for us and the future of the planet.

### Increasing human control over AI will reduce harm (Claim 5)

One of the underpinning goals of AI development is to transfer greater independence to AI because of the supposed benefits it could bring. The commonly held assumption within the AI field is that to get greater automation, we must give away control to AI. Conversely, higher levels of control hinder automation (Pacailler et al. 2022). HCAI states that this is misleading and we can have high levels of automation and control (Shneiderman 2022). We need *more* human control over AI decision-making, not less (this is also reflected strongly as 'human oversight' in High-Level Expert Group on AI 2019). HCAI advocates state that AI can and should be developed with *high levels of human control* and *high levels of automation* (Shneiderman 2022, p. 9). We do not need to ‘sacrifice human control when incorporating higher levels of automation’ (Pacailler et al. 2022, p. 471). We need to place a greater emphasis on the ability and skills of humans, particularly, when we are the ones most impacted by AI decision-making (Shneiderman 2022).

Being in control of AI is one of the foundational goals of HCAI (Schmidt 2020). Fundamentally, HCAI promotes AI that is humanly controlled (Xu and Dainoff 2023). Humans should be included throughout the AI process (He et al. 2021) to ensure people ‘feel safe and have self-determination’ (Schmidt 2020, p. 1). One of the ways to do this is by ensuring a human-in-the-loop process for better accountability and to reduce harmful biases (Mhlanga 2022). The human-in-the-loop approach is the process of involving humans in the intensive training, testing, and tuning of machine learning models. Humans can categorize the training data to help the model understand which characteristics to identify, for example. People may, again, evaluate the quality of the model’s prediction, as well as provide input to the algorithm when it gets something wrong, implying that people are a component of the model’s continuous feedback loop (Mhlanga 2022, p. 15).

We must be able to benefit from AI, while still maintaining control of it (Shneiderman 2022). Because AI cannot understand humans as well as we understand ourselves (Riedl 2019, p. 34), AI will make errors along the way; for example, causing harmful biases against people and groups (He et al. 2021). These errors are usually caused by harmful biased data and how the algorithms are designed or interpreted/used (Lepri et al. 2017). HCAI aims to ensure that humans are not harmed by AI through harmful biases (Xu and Dainoff 2023). HCAI advocates claim that AI may cause harm to humans if there is not enough adequate human supervision (Xu et al. 2022). Therefore, we need to increase human oversight of AI to reduce these harmful biases (Shneiderman 2022). If humans are involved in the process, they can better monitor AI and reduce harmful biases (Pacailler et al. 2022).

HCAI proponents state that harmful AI bias results from bias in (human) data being fed to the AI, the way that algorithms are designed (by humans), or how AI’s results are understood and implemented (by humans) (Lepri et al. 2021; Ozmen Garibay et al. 2023; van Berkel et al. 2022). There seems to be a logical inconsistency within the HCAI mode of thought here: AI will cause harmful biases because of biases in human data, or from the human AI engineers’ biases when developing it, or how humans interpret its results; *but*, we should increase human involvement in the AI process to reduce these harmful biases (which are largely caused by humans).

Instead of simply repeating our mistakes, maybe we should take a step back and identify some of the underlying *causes* of these biases in the first place. One of the underpinning reasons for many of these biases is the effort and push to categorise, classify, and pin down human subjects within an array of clusters and nodes of what defines the human or a particular type of human. This was an issue that Foucault tried to address in his work as he challenged those trying to distinguish the human through a series of characteristics. For Foucault, we are much more than just the composition of certain traits or behaviours; as every person is the entire collection of feelings, emotions, and experiences, as well as one’s body and its flow and interaction within the world (May 2014, p. 10).

Within these algorithmic approaches to defining the human, they implicitly attach some idea or definition of the human and some unchanging feature(s) that defines it (Paden 1987; Sutherland and Patsoura 2015). However, Foucault would reject such approaches that claim that human has some essential boundless characteristics that define them (Chomsky and Foucault 2015; Lightbody 2010; Wilkin 1999). We need to be critical of approaches that limit the subject to human universals or assumptions about the inner essences of humankind (Pyyhtinen and Tamminen 2011).

Foucault stated that modernity’s focus on the human sciences is mainly used as a way to categorise and classify the human, focusing on human life (biology), labour (economics), and speech (linguistics) (Foucault 1994; Pyyhtinen and Tamminen 2011). However, Foucault could not have foreseen the sheer extent of algorithmic datafication that humans are witnessing today. The mode of classifying and evaluating the human within AI processes has become vastly more dense and all-encompassing since Foucault’s time. AI has accelerated and amplified these classifications as a result of the abundance of data available on individuals [for example, ‘Facebook [alone] can classify roughly 52,000 traits of each of its users’ (Green 2018)]. The eagerness to increase algorithmic subjectification creates specific boundaries, sub-categories, and silos, of different characteristics, habits, and traits that define us and divide us (cf. Zuboff 2019).

The human is categorised, classified, and boxed (as the result of AI) into countless categories, sub-categories, and sub-sub-categories, of what it means to be a particular type of human. While it is claimed that this brings many benefits, such as greater identification of individual needs, tailoring relevant ads to individuals, and providing more suitable online content, it also poses great threats and harms to individuals, such as causing harmful bias/discrimination, harmful nudging, and creating digital echo chambers or filter bubbles (Benkler et al. 2018; Möller 2022; Scharkow et al. 2020). These issues are fundamentally based on trying to define, narrow down, and categorise the human. Harmful biases are often caused by trying to identify and group individuals and by allocating them to specific classifications or segments of humanity, rather than by an insufficient level of human control in the process (as HCAI proponents claim).

Foucault examined different types of oppositions to identify common struggles in society between individuals and groups. These were the ‘opposition to the power of men over women, of parents over children, of psychiatry over the mentally ill, of medicine over the population, of administration over the ways people live’ (Foucault 1982a, b, p. 781). These struggles can also be contrasted with some of the oppositions seen in the field of AI ethics: the opposition to the power of AI companies over how to classify groups of people, the opposition that states use AI to surveil and control citizens, and (in the context of HCAI) the opposition that AI developers do not give enough attention to the human.

An underpinning theme of the oppositions that Foucault analysed is that they ‘are a refusal of these abstractions, of economic and ideological state violence, which ignore who we are individually, and also a refusal of a scientific or administrative inquisition which determines who one is’ (Foucault 1982a, b, p. 781). While written forty years ago, this analysis can also be compared to the foundational issues underpinning AI debates; namely, the refusal to permit economic and ideological abstractions of individuals, and the scientific and administrative inquisitions of AI that attempt to determine and classify who one is.

Claiming that AI development is not human-centred enough perpetuates the abstraction of the individual within the bounds of what is defined as *human*. It fails to tackle some of the underlying problems within AI development. HCAI proponents find fault with AI companies, developers, or the industry for not placing humans at the centre, but they fail to look at or challenge the root cause of many of these issues. While asking more from particular groups in AI; we also need to look at the ideologies underpinning AI in a critical way. This point can be illustrated in a quotation from Foucault (with my inclusion of how it can be applied to the AI debate in square brackets):

‘…the main objective of these struggles is to attack not so much "such or such" an institution of power [e.g., the AI industry], or group [e.g., AI developers], or elite [e.g., AI companies], or class but rather a technique, a form of power [e.g., the classification and categorisation of individuals through the use of AI]’ (Foucault 1982a, b, p. 781).

The technique or form of power in the context of AI attempts to classify, categorise, and define individuals as a way of dictating what that individual clicks on, likes, buys, thinks, and votes. It is not confined to one particular application of AI. Still, instead, the underlying mentality pervasive within the AI field is that individuals are simply the identification and categorisation of their behaviours and patterns that are defined by the data points that they digitally interact with within the world. This technique of power ‘applies itself to immediate everyday life which categorizes the individual, marks him by his own individuality, attaches him to his own identity, imposes a law of truth on him which he must recognize and which others have to recognize in him’ (Foucault 1982a, b, p. 781).

The individual is categorised and grouped through AI classifications. They are marked by their individuality, captured by AI through our constant use of search engines, social media, and online purchases; this is then attached to our digital identity, which is then imposed back upon us by telling us who we are (or who we should be) through our algorithmically-defined life. Therefore, simply advocating for greater control of AI or placing humans at the centre does not necessarily solve the issues caused by the reduction of individual subjects to a cluster of nodes and data points. To tackle these issues effectively, one needs to not only address the resultant outcomes of harmful AI solutions (such as harmful bias) but also, the causes that underpin and allow these harms to occur in the first place. The HCAI approach and most AI ethics approaches for that matter, tend to focus on surface-level issues prevalent within AI. While this is certainly admirable and is better than doing nothing, they fail to look at many of the much larger and underpinning *causes* of these issues.

## Conclusion

This paper focused on the HCAI framework and evaluated five common claims in the literature endorsing this position. It was shown that HCAI advocates regularly refer to ‘machine-centred’ approaches by which they create HCAI in response. Machine-centred approaches, and the need for HCAI in response to these positions, is that they are inherently unhuman, opposed to humans, or against human values. However, AI is being developed by humans and for humans. Nobody endorses or exemplifies the machine-centred approach to AI that HCAI passionately opposes, which points to a ‘strawman argument’ made by HCAI proponents. While there are many concerns about the impacts caused by AI, framing the debate in such a dichotomous and polarising way as ‘human-centred’ vs ‘machine-centred’ is also misleading and provides an overly simplistic narrative of what is taking place in AI.

In addition, HCAI has created an in-built critical mechanism that forces people to endorse it. For example, if you do not support it, you are simply a nihilist or misanthropist. Similarly, in another sense, HCAI provides a way for corporations to get behind an approach that is seen as ethical and progressive while having very few requirements or demands being placed on them to initiate. HCAI is a form of ethics-washing (or ‘human-washing’) that large tech companies can easily claim to endorse, while not being pressured to demonstrate how they implement such an approach.

While HCAI proponents promote the view that adopting AI is inevitable and ‘resistance is futile’ (which, as seen above, also extends to the adoption of the HCAI framework), we should be wary of such positions as they restrict our decision-making capacities and ability to counter, resist, and refrain from certain courses of action. While many technologies have brought great benefits to society, there are also many examples of the opposite (think CFCs, DDT, Agent Orange, and the hydrogen bomb), and there is no guarantee that AI, in all its compositions, applications, and uses, will automatically bring a more significant net benefit to humanity. Therefore, we should be skeptical toward the idea (as found with HCAI) that AI will automatically lead to ‘progress’.

However, if one still wants to accept the deterministic view of our future illustrated in HCAI (that AI is here to stay, and we have to get used to it), this does not necessarily mean we should endorse a view that only cares for humans. As Karl Marx once commented, ‘History repeats itself, first as a tragedy, second as a farce’ (Marx 1963). Maybe we need to take a step back and ask ourselves: do we need a more *human-centred* approach to anything when we see the catastrophic events caused by taking such an anthropocentric stance throughout history (e.g., environmental destruction (United Nations 2019), biodiversity loss (United Nations 2023), and global climate change (IPCC 2020; Pachauri and Reisinger 2007)). Maybe Ben Shneiderman was right—we do need another Copernican Revolution. However, perhaps a Copernican Revolution reconsiders our place in the world and asks if AI can be used as a source of good, to right some of the wrongs we have caused, and to include considerations for other species and the planet as well as ourselves.

### Limitations and future research

A criticism against Foucault is that he provides no appealing alternative to humanism (Paden 1987). While his work in *The Order of Things* can provide interesting insights into how we can critique HCAI and identify many flaws within such an approach, there is little clear-cut guidance on where we go from here. Foucault's characterisation of how humans have tried to modify themselves throughout history, which he discussed in Technologies of the Self (Foucault 1988b, 1990), may provide a more concrete approach for developing a Foucauldian ethics of AI.

In addition to Foucault’s early period, encapsulated through his archaeological approach, his subsequent works on power-knowledge relations and apparatuses would provide further insights into why and how the dividing practices found within AI occur. Further reflections on Foucault’s genealogical and ethical work in his mid to late career would also provide a rich contextualisation to how algorithmic dividing practices represent a disciplining over the subject (Foucault 2012), the power/knowledge relations implicit and explicit within AI (Foucault 1980), and also resistance to this (Foucault et al. 2008; Giraldo Díaz 2006; Pickett 1996).

Foucault’s *The Order of Things* could also be useful for examining how AI has historically developed. In *The Order of Things*, Foucault examines how different historical epochs are defined within epistemes, namely, how structures of thought are shaped and knowledge is created, defined, and understood. Foucault’s text could be used to help develop a specific examination of how different types, understandings, and applications of AI have historically developed and what this implies for society (e.g., from rule-based systems to machine learning to deep learning and so forth). This analysis of our understanding and interaction with AI in different eras may offer exciting insights into humankind’s interaction, involvement with, and creation of AI. While this paper has touched upon this in the context of one of the latest iterations of AI, namely HCAI, a Foucauldian analysis of the different shifts in the fundamental structures of thought about AI over time would offer a fruitful contribution to the debate. All of this analysis could support and contribute to the debate surrounding human interaction with AI, human intelligence, and how this may look in the future.

## Data Availability

The author confirms that all data generated or analysed during this study are included in this published article.

## Footnotes

1 The search query string was used on February 1st 2023: (ALL ("human-centred AI") OR ALL ("human-centered AI") OR ALL ("human-centric AI") OR ALL ("human-centred artificial intelligence") OR ALL ("human-centered artificial intelligence") OR ALL ("human-centric artificial intelligence")). 1639 were published since 2019.

2 While his works after *The Order of Things* would also certainly be interesting to analyse in the context of AI, it is beyond the scope of this paper. In addition, it would blur and conflate Foucault’s ideas if one were to attempt to capture his entire body of work within one paper on AI. As Foucault explicitly stated himself, do not expect him (and his work) to remain the same (Foucault 1982a). Much of the literature about Foucault and technology concentrates on his later periods of writing, so while they offer interesting findings and views, for the most part, they were only used sparingly in writing this paper. The Order of Things illustrates Foucault’s approach to the human and the modern episteme most effectively and this offers the most evident critique of HCAI, which emphasises the human-centring of AI.

3 During the Renaissance episteme (up to the end of the sixteenth century), scientific investigations and knowledge mainly focused on resemblance, whereby language is used to represent things. Foucault viewed the episteme as being one that focused on resemblance in the construction of knowledge development in Western culture (Foucault 1994). Resemblance guided the interpretation of texts, organised symbols and their meaning, and determined the way of representing them (Foucault 1994, p. 17). To search for meaning meant identifying resemblances, similarities, and likenesses (Foucault 1994, p. 29). One example of this resemblance is through language and a sign of things in the world: ‘The names of things were lodged in the things they designated, just as strength is written in the body of the lion, regality in the eye of the eagle’ (Foucault 1994, p. 36). Even with apparent differences, such as varying languages, there is unity in the manner that they are expressed and what they convey. ‘Knowledge therefore consisted in relating one form of language to another form of language; in restoring the great, unbroken plain of words and things; and making everything speak’ (Foucault 1994, p. 40). However, the seventeenth century saw a change from resemblance toward representation (the emergence of the classical period). Representation emerged out of when ‘the relation between the sign and what it signifies is no longer a natural one’ (May 2014, p. 47).

4 The binary interaction of resemblance between sign and object is broken within the classical episteme of representation and order (May 2014, p. 48). The signs of objects were no longer something we must decipher the hidden meaning of, but instead, they are representations of things that we must place in a proper order so that we can understand them (May 2014, p. 48). Order is the way in which we structure things (Foucault 1994). There can be disagreement about the claims and ways of structuring, but this must be within the parameters of the ordering of representation (May 2014, p. 49). Ordering, within the classical period, begins with observation of things in the world, which is used to create order out of these observations (May 2014, p. 49). Language provides us with the necessary tools to order these objects. The classical episteme focused on taxonomies, classifications, and subdividing all aspects of life and the world into manageable representations (Foucault 1994). During the classical period, mathesis and taxinomia prevailed as mathematical systems and methods of classification of things that were similar but differed ‘on variation, measurement and number’ (Downing 2008, p. 43).

5 Foucault uses the term man, which would have traditionally been used in the past. Man is an archaic term that has been used in philosophy to refer to the whole of humankind, but is obviously limited in its inclusion. However, for the purpose of this paper, I will refer to the human, instead of man.

6 The human is both ‘the knower and the known, and must constantly oscillate between the two. Man is at once the subject and the object of knowledge’ (May 2014, p. 53).

7 While past epistemes indeed analysed people and ‘human beings’, and gave them importance in the world, they could not conceive of the human in the same manner as emerged in the Modern episteme (Foucault 1994, p. 318).

8 And it did this throughout three important human sciences: economics, biology, and linguistics. These three disciplines, for Foucault, formed the basis of the modern project of knowledge (Foucault 1994). They evaluated the concepts of life (biology), labour (economics), and language (linguistics), which were fundamental for human knowledge (Foucault 1994). These ways of examining the world were no longer focused on the order of the visible, but more so, on the ‘underlying structure or nature’ of everything (May 2014, p. 51).

9 In Foucault’s own words, the point of writing should work as a way ‘of creating a space into which the writing subject constantly disappears’ (Foucault 1984a, p. 102). This paper will use Foucault’s texts in a similar way, applying them to the topic of HCAI, while allowing the author to disappear in the process. One must deprive the subject and its role as the originator of a text (Foucault 1984a, p. 118) and permit texts to be modified and used in different ways. Foucault makes this point in relation to Freud and Marx: ‘reexamining Freud's texts modifies psychoanalysis itself, just as a reexamination of Marx's would modify Marxism’ (Foucault 1984a, p. 116).

10 Both authors are also referenced in other HCAI texts (Schmidt 2020 refer to Engelbart, and Xu et al. 2022 refer to Licklider).

11 While humans as a species or genus were analysed before modernity, but not the socially-constructed notion of the human (Foucault 1994, p. 309).

12 This paper is not making the claim that Copernicus was the first person to acknowledge or claim that the sun was the centre of the universe, as there are many examples throughout history of those proposing this ‘heliocentric’ model of the universe; for example, there is evidence that as far back as the third century BC, Hellenistic authors such as Aristarchus of Samos, wrote about it. This paper is instead sketching out the views of Shneiderman about the Copernicus Revolution.

13 This is not to say that this is necessarily the only, or correct, interpretation of the Copernican Revolution. Some (Danielson 2001) claim that using Copernicus’ work to disprove the anthropocentric (geocentric) worldview is misleading. Danielson makes the point that it is incorrect to use this understanding of the Copernican Revolution and its non-anthropocentrism in a literal way (however, it can be used in a figurative way, which is the way Shneiderman intends it. He states it is an ‘extreme metaphor’ (2021, p. 113)).

14 The function of centring AI development around the human (in HCAI) appears to be an *ethical* one to ensure the autonomy of individuals, cultivate human welfare, and protect privacy. The function of Foucault’s work in The Order of Things was to evaluate what is seen as acceptable discourse and the epistemic assumptions underpinning this discourse (Foucault 1994). Despite this, Foucault’s analysis of the human and its impact on human science discourse in the modern episteme may offer valuable insights about HCAI’s human-centring, particularly its starting premise that the human is not currently at the centre of AI development.

15 This view stems from the field of human-centred design, which claims that designers have given too much value to a product's function rather than concentrating on the needs of the end user; cf. Hanington, 2010.

16 It is certainly not non-human species or the environment, as these have been mostly excluded from the AI debate until very recently (e.g., Hagendorff et al. 2022; [author, 2022]; Singer & Tse 2022). In Jobin et al.’s (2019) analysis of 84 AI ethics guidelines, only five guidelines mentioned either ‘sentient creatures’ (Montreal Declaration 2017), the ‘planet’ (Tieto 2018; UNI Global 2017) or the environment (European Group on Ethics in Science and New Technologies 2018; IEEE 2019) (and are usually only referred to because they are of value to humans). The HCAI literature is no different, with the environment being understood as only instrumentally valuable for *human flourishing* (Ozmen Garibay et al. 2023, p. 395) and *economic growth* (Ozmen Garibay et al. 2023, p. 413). Therefore, the development of AI is not being done for the benefit of non-human species or the environment.

17 Conducting web browser searches for the term machine centred approach to AI or machine centred AI only brought back pages and pages of articles about HCAI.

18 As of April 14th, 2023.

19 However, this open letter, and those who signed it, were criticised as using it as a red herring to deter away from current issues prevalent within AI. Critics claimed that the open letter prioritised ‘imagined apocalyptic scenarios over more immediate concerns about AI—such as racist or sexist biases being programmed into the machines’ (Paul, 2023). While others (such as Elon Musk) signed this open letter, only to shortly afterwards invest heavily in AI start-up xAI (Clayton 2023).
